# Novel use of a single port laparoscopic surgery device for minimally invasive pancreatic necrosectomy

**DOI:** 10.1308/003588412X13373405386015

**Published:** 2012-09

**Authors:** D Subramaniam, WK Dunn, J Simpson

**Affiliations:** Nottingham University Hospitals NHS Trust,UK

## ERRATUM

The following article appeared on page 372 of the July 2012 issue of the Annals. Unfortunately the images that accompanied the tip were incorrect. We reproduce the image below with the correct images displayed. The Editor apologises for any confusion caused.

## BACKGROUND

The development of pancreatic necrosis is a significant complication of acute pancreatitis and can result in progressive multiple organ failure and death. Recently, in an attempt to reduce the high morbidity and mortality from open necrosectomy, minimal access techniques have been developed.^1^

## TECHNIQUE

With the recent advent of single port laparoscopic surgery, a single access port (SILS™; Covidien, Mansfield, MA, US) can be used to gain retroperitoneal access (Fig 1) and allow necrosectomy to be performed. During the procedure, irrigation with warmed 0.9% saline or low CO_2_ pressure (8mmHg) permits visualisation of the retroperitoneum, and standard laparoscopic graspers and a suction device can be placed through additional port sites in the unit to allow removal of necrotic tissue (Fig 2). Post-operatively, continued irrigation of the retroperitoneum is maintained at 100ml/hr with 0.9% saline. We have used this technique successfully in three patients.

**Figure 1:**
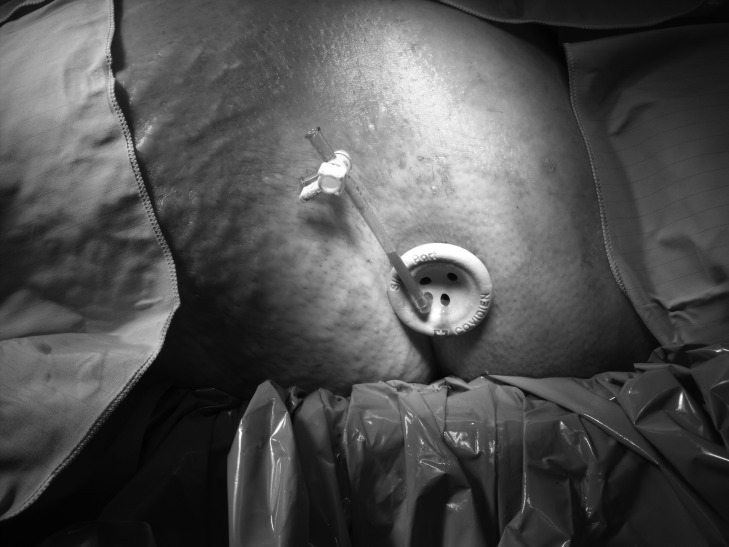
SILS™ port allowing access to the retroperitoneum

**Figure 2:**
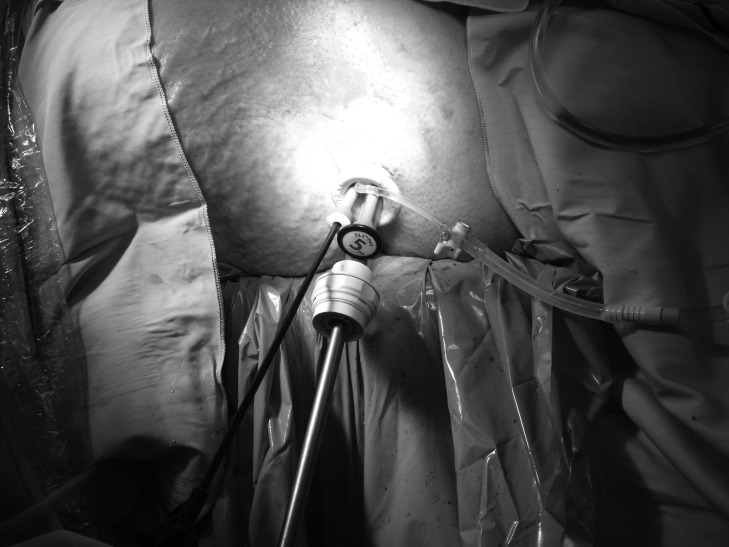
Placement of laparoscopic grasper and suction device in the single access port

## DISCUSSION

The technique of minimally invasive necrosectomy has been well described previously^2^ and has been shown in certain situations to have advantages over the traditional open approach.^3^ This relatively standard technique employs the use of an operating nephroscope. The advantage of the SILS™ port is that standard laparoscopic instruments can be used and if the retroperitoneal cavity is large, two laparoscopic graspers can be used simultaneously for tissue debridement.
